# Harnessing ADAR-Mediated Site-Specific RNA Editing in Immune-Related Disease: Prediction and Therapeutic Implications

**DOI:** 10.3390/ijms25010351

**Published:** 2023-12-26

**Authors:** Shenghui Weng, Xinyi Yang, Nannan Yu, Peng-Cheng Wang, Sidong Xiong, Hang Ruan

**Affiliations:** 1 Jiangsu Key Laboratory of Infection and Immunity, Institutes of Biology and Medical Sciences, Soochow University, Suzhou 215123, China; shenghui.weng14@alumni.xjtlu.edu.cn (S.W.); ryrl970311@gmail.com (P.-C.W.); 2 MOE Key Laboratory of Geriatric Diseases and Immunology, Suzhou Medical College of Soochow University, Suzhou 215123, China

**Keywords:** ADAR, RNA editing, immune-related disease, computational resources

## Abstract

ADAR (Adenosine Deaminases Acting on RNA) proteins are a group of enzymes that play a vital role in RNA editing by converting adenosine to inosine in RNAs. This process is a frequent post-transcriptional event observed in metazoan transcripts. Recent studies indicate widespread dysregulation of ADAR-mediated RNA editing across many immune-related diseases, such as human cancer. We comprehensively review ADARs’ function as pattern recognizers and their capability to contribute to mediating immune-related pathways. We also highlight the potential role of site-specific RNA editing in maintaining homeostasis and its relationship to various diseases, such as human cancers. More importantly, we summarize the latest cutting-edge computational approaches and data resources for predicting and analyzing RNA editing sites. Lastly, we cover the recent advancement in site-directed ADAR editing tool development. This review presents an up-to-date overview of ADAR-mediated RNA editing, how site-specific RNA editing could potentially impact disease pathology, and how they could be harnessed for therapeutic applications.

## 1. Introduction

ADAR (Adenosine Deaminases Acting on RNA) is a deaminase family specifically targeting adenosine in the double-stranded RNAs [1], which replaces an amino group of adenosines with a keto group, thus contributing to the adenosine to inosine (A-I) conversion. Inosine is further recognized as guanosine (G), which has a similar structure to inosine. This conversion forms a wobble RNA loop or leads to A-G RNA substitutions during further processes [2,3,4].

ADARs are presumed to originate in the early ancestors of metazoans because they are conserved in most major phyla of extant metazoans. A-G substitutions are far more dominant in these species than the other 11 possible nucleotide substitution types [5]. ADAR-mediated A-I RNA editing sites (RESs) are abundant in eukaryotes and mainly in the nascent nuclear mRNA [6]. Across 16 representative metazoan species, ADAR-mediated RNA editing is generally conserved [7], and these post-transcriptional RNA editing events could be considered the hallmark of the metazoan transcriptional regulation [8,9]. In mice and humans, large-scale A-I RNA editing sites are observed in the non-coding regions, while a few editing sites occur in the coding region, such as conserved recoding sites of FlnA, CyFip2, Blcap, and IGFBP7 [10]. In Bilateria, the ADAR-edited recoding sites tend to accumulate in neural and cytoskeleton genes [5].

Abnormal expression of ADAR has been detected in numerous diseases, such as multiple autoimmune diseases and human cancers, and the abnormal expression of ADAR1/2 was positively correlated with the degree of RNA editing [11]. Apart from the pathogenetic global changes in editing levels, many specific RNA editing sites play opposite roles at various stages of cancers; some are strongly associated with high aggressiveness and poor tumor prognosis [12,13,14,15], while some RESs are related to tumor suppression [16,17]. In addition to non-synonymous substitutions on coding sequences [18], there are other types of pathogenic editing sites, such as RESs on 3′ untranslated region (UTR) [19], intron [13], and microRNA (miRNA) [20]. RES is also associated with anti-tumor drug resistance [18]. In the context of human genetics, many editing quantitative trait loci (edQTLs) were significantly enriched in genome-wide association study (GWAS) signals for autoimmune and immune-mediated diseases. RNA editing sites near these edQTLs often exhibited a reduced editing state in disease-associated samples, which might activate the melanoma differentiation-associated gene (MDA5) pathway-mediated interferon response [21,22]. Furthermore, RESs are also believed to contribute to the pathogenesis of other immune-related neurodegenerative diseases, including schizophrenia [23].

To further study the functions of ADAR-mediated RNA editing, the broad spectrum of RNA editing sites is necessary. Developing computational tools that efficiently predict RNA editing sites across the transcriptome is now plausible using high-throughput sequencing technology [24,25,26]. Large-scale RNA editing sites have been identified in both human tissues and animal models, thus providing ample opportunity to explore site-specific editing functions [27,28,29].

Recently, there has been growing interest in recruiting endogenous ADAR for in vivo engineered site-directed RNA editing (SDRE) [30,31]. Exogenous ADAR was also employed to operate precise editing. Along with A-I editing, engineered C-U editing has also been developed [32]. Based on the understanding of the recognition and regulation mechanism of ADAR enzymes, various ADAR recruitment methods have been adopted to improve the efficiency of SDRE, including adding hairpin structures, R/G motifs, or introducing Cas13 proteins to guide RNAs [33].

In this review, we first introduce the concept of ADAR-mediated, pattern-recognized RNA editing and its critical relevance to immune-related pathways. We further explore the regulatory rules of site-specific editing in maintaining cellular homeostasis and the effects of disease-related dysregulated RNA editing events. We also highlight recent computational approaches that predict transcriptome-wide RNA editing sites from high-throughput RNA sequencing data. We discuss the identification of site-specific RNA editing and its editing frequency using various prediction tools. In addition, we present interactive web servers for functional analysis of RNA editing sites and data resources that contain large-scale RESs detected in human and animal models. The last section discusses the laboratory applications and therapeutic potentials of engineered site-directed ADAR editing.

## 2. ADAR Proteins as Pattern Recognizers in Immune-Related Pathways

### 2.1. ADAR Proteins Are Cross-Species Conserved Pattern Recognizers

In mammals, ADAR enzymes have three main subtypes: ADAR1, ADAR2, and ADAR3 [34]. ADAR1 and ADAR2 are expressed in multiple organs, while ADAR3 is mainly expressed in the brain [9]. ADAR1 is widely expressed almost throughout the human body except for skeletal muscle. Although ADAR1 and ADAR2 are expressed in most tissues, the identified edited pre-mRNA typically encodes receptors in the central nervous system [35]. ADAR2 exhibits exceptionally high expression in the central nervous system. It is thought to be primarily responsible for site-specific editing of adenosine in shorter RNA hairpins of central nervous system transcripts [36]. In contrast to ADAR2, which is located mainly in the nucleus, the p150 isoform of ADAR1 can shuttle between the nucleus and cytoplasm by binding to transportin-1 or exportin-5 [8,35,37,38,39]. The translocation of p110 was not as regular as p150 but was observed under stress to block the stress-induced Staufen1-mediated mRNA degradation [40,41]. Intracellular localization analyses of ADAR revealed that ADAR1 and ADAR2 were dynamically moving in nuclei in vivo. The transient sequestration of ADAR1 and ADAR2 in the corners of the nucleus may be caused by the rich endogenous dsRNA structures from the small nucleoli RNA in these corners. In contrast, ADAR homologous protein ADAT (primarily edited tRNA), which lacks a dsRNA binding domain, has not been observed to accumulate dynamically in the nucleus [38]. To activate the A-I RNA editing activity, the homodimerization of ADAR1 and ADAR2 is required [42]. Mammalian ADAR3 is catalytically inactive and acts predominantly as a dsRNA binding protein. ADAR3 is a brain-specific protein and might work as an inhibitor to regulate the process of ADAR1/2-mediated RNA editing [43,44]. According to some studies, the expression of ADAR1 or ADAR2 alone did not correlate with the editing level. In contrast, the combined value of ADAR1 + ADAR2-ADAR3 was positively correlated with the editing level of RNA [45]. 

The ADAR enzymes exhibit a regional preference to catalyze specific sites by recognizing nearby sequences, as confirmed in *Drosophila* and humans [46,47,48]. ADAR1 and ADAR2 catalyze their preferred characteristic dsRNAs [45,49,50]. There is no significant difference between ADAR1 and ADAR2 in the global editing sites or regional preference [50]. The values fluctuated slightly between studies, but introns or 3′UTR always cover the most editing sites, and 5′UTR or coding sequences account for only a tiny fraction [50,51]. In humans, the RNA editing sites (more than 80%) are dominantly located in repeat regions, such as short interspersed nuclear elements (SINE), long interspersed nuclear elements (LINE), long terminal repeats (LTR), etc. Only a tiny fraction of RNA editing sites happened on exons. Globally, most of these sites occur in primate-specific, small 140–300 bp Alu elements—a class of repeating SINE (short interspersed nuclear element) inverse elements. The Alu element covers the most editing sites relative to other repetitive elements in humans. Alu was observed mainly in introns and 3′UTRs, which are regions of the RNA molecule that are not translated into protein sequences but have regulatory functions [52,53,54,55]. This shows that ADAR1 primarily edited the Alu element in mRNA transcribed by RNA polymerase II, but not the putative pol-III-transcribed Alu elements [55]. In addition, although both ADAR1 and ADAR2 enzymes target dsRNA hairpins and there are some overlapping editing substrates between ADAR1 and ADAR2 in cells expressing both of these two enzymes, ADAR1 is particularly biased towards catalyzing around 300-base-long hairpins formed from paired inverted copies of Alu elements in the pre-mRNA [10]. ADAR1 has also been proven to contribute to hyper-editing in the repeat element, and ADAR2 tends to be responsible for non-repetitive coding sites [45], which may be induced by the formation of the sense–antisense structure on these non-repeat regions [5,56]. 

### 2.2. Immune-Related Pathways Mediated by ADARs

**Figure 1:**
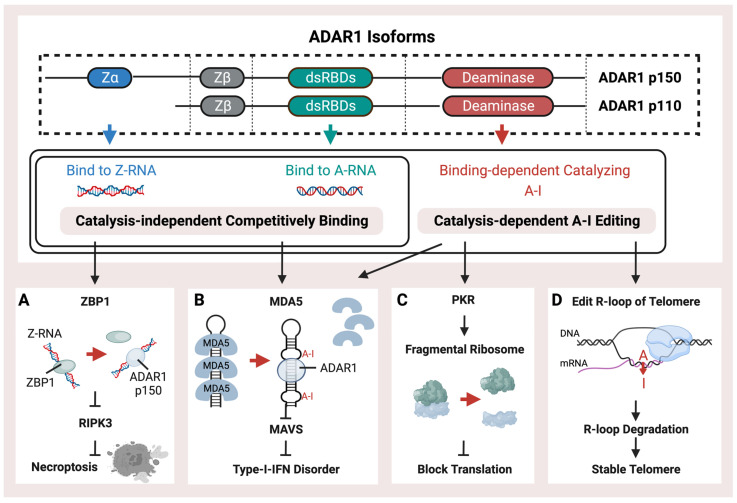
Schematic representation of the structure and functions of ADAR1. ADAR1 isoforms, p110 and p150, possess a right-handed A-RNA bindable double-strand RNA binding domain (dsRBD) and a catalysis domain (deaminase). p150 also contains an additional left-hand Z-RNA binding domain (Z-alpha). ADAR1 performs catalysis-independent competitive binding or catalysis-dependent A-I RNA editing through its distinct binding and catalysis domains. ADAR1 has multiple pathways of operation, (**A**) including binding with endogenous Z-RNA to inhibit RIPK3-induced necroptosis and to block the activation of ZBP1. (**B**) Additionally, ADAR1 prevents immunogenetic dsRNA from inducing MDA5-mediated Type-I interferon disorder by either binding or editing to impede the recognition of dsRNA. (**C**) Moreover, ADAR1-mediated RES inhibits the PKR pathway, thus facilitating translation shutdown. (**D**) Finally, ADAR1 catalyzes the R-loop to promote its degradation and stabilize the telomere.

ADAR1 is mainly present as two isoforms, ADAR1 p150 and ADAR1 p110, which are named based on their molecular weight (Figure 1). ADAR1 p150 is 150 Da in size and is crucial for protecting mammals against viral infections. It serves as a major pattern recognition protein. It is induced by interferon [57], thus emphasizing its importance in the body’s immune response against viruses. Both the p150 and p110 isoforms can be found in the cytoplasm. ADAR1 p110 is a shorter isoform with a 110 Da size. These two ADAR1 isoforms are generated from alternate splicing. They share a double-strand RNA binding domain (dsRBD) and a deaminase [58]. The double-strand RNA binding domain (dsRBD) shared by ADAR1 p150, p110, and ADAR2, primarily recognizes common right-handed dsRNA (A-RNA) conformations. Type-I-Interferon-inducible p150 has an additional Zα (Z-RNA binding domain, ZBD) at the N-terminal compared to p110. Thus, p150 could bind endogenous Z-RNA to avoid activating ZBP1 and downstream RIPK3-mediated necroptosis by recognizing unbound Z-RNA (Figure 1A), thus mediating the innate immune system activation caused by immunogenetic Z-RNA [59,60,61,62,63]. It has been discovered that ZBP1 plays a crucial role in sensing Z-RNA during influenza A virus infection. This activation of ZBP1 leads to hyperinflammation and necroptosis, which are some of the main ways the body fights off viral infections [64]. When ADAR1 cannot prevent the binding of ZBP1 to Z-form RNA through editing or binding, activated ZBP1 activates downstream Receptor Interacting Serine/Threonine Kinase 3 (RIPK3)-mediated necroptosis. The ZBD of ADAR1 p150 is primarily bound to endogenous left-handed dsRNA (Z-RNA) enriched in the 3′UTR of the interferon-stimulated genes (ISGs) [59]. ADAR1 p150, unlike ADAR1 p110 and ADAR2, can recognize Z-RNAs and inhibit downstream immune responses by independently binding (Figure 1). Overexpressed ZBD could rescue the phenotype caused by the lack of a catalysis domain. In diseases, aberrant ADAR1 p150 may be involved in ZBP1-mediated cell necrosis. Activation of the ZBP1 pathway may also be inhibited by p150, thus representing a potential treatment option in cold tumors [59]. Different Alu families contain a conserved Z-forming sequence [65]. The Z-forming sequence is prone to forming Z-DNA conformations and could be recognized by the ZBD of ADAR1 p150 [65,66,67]. Different Alu families contain a conserved Z-forming sequence. The Z-forming sequence is prone to forming Z-DNA conformations and could be recognized by the ZBD of ADAR1 p150 [66]. In addition, p150’s extra RNA binding domain allows for higher binding ability than p110, resulting in more editing sites [51]. Except for the Z-forming sequence, other RNA modifications can potentially affect the binding between ADAR and Alu, such as 2′-O-methyl and N6-methyl adenosine marks. Because these modifications share the same substrate with ADAR, exogenous double-stranded RNA, they may have a competing relationship with ADAR-mediated RNA editing and play similar roles in regulating MDA5-mediated immune pathways [68,69].

ADAR-mediated RNA editing is a crucial mechanism in human cells that plays a key role in fighting against viral infections. This mechanism helps to differentiate between endogenous and exogenous double-stranded RNA, which is essential for the immune system to recognize and respond effectively to viral threats. Editing of dsRNA through co-transcriptional ADAR-mediated A-I editing prevents downstream Type-I-Interferon activation of MDA5 (Figure 1B) by creating wobble A-I base pairs so that MDA5 cannot recognize endogenous dsRNA [51,54,70]. ADAR is critical during mice’s embryonic development to maintain hematopoiesis [71,72]. The mice lacking ADAR1 died at the embryonic stage with wide apoptosis in multiple tissues, fetal liver disintegration, and defective hematopoiesis, and mice with homozygous ADAR1 p150 deletion exhibit a similarly lethal phenotype [73,74,75]. The MDA5 pathway induced by loss of ADAR1 function is associated with the interferon (IFN) disorder of developing AGS [76,77]. 

It has been demonstrated that mice can tolerate the loss of global A-I editing when the type-I IFN-induced pathway is blocked by MDA5 inhibition, thus indicating that ADAR1-mediated RNA editing sites are not crucial for the development and homeostasis of mice [78,79]. As a result, the MDA5-dependent interferon pathway, induced by unedited Alu elements, could be the main feature of ADAR and probably plays a significant role in interferon-related diseases. Both loss of function (LOF) mutations on ZBD or dsRBD of ADAR1 have been identified in AGS patients [76,80]. The catalysis-independent competitive binding of ADAR proteins against MDA5 may also achieve the same effect as the A-I editing (Figure 1B) [81]. In addition to the MDA5 activated by un-catalyzed dsRNA, the Z-RNA binding ability could also inhibit the MDA5 pathway, and the LOF mutation, such as P154A or W197A on ZBD of ADAR1, could activate the MDA5 pathway without decreasing the global RNA editing level [82,83]. Meanwhile, other dsRNA sensors, such as OAS and RIG-I, acted as the immune activators when the ADAR editing was insufficient [84,85]. 

During the Type-I-IFN response, the Protein Kinase R (PKR) pathway (Figure 1C) could also be activated to block translation by the endogenous dsRNA through the fragmentation of the ribosome. Knockdown of ADAR1 caused differentiated human neuronal progenitor cells to exhibit both MDA5-activated interferon upregulation and PKR activation, accompanied by cell death [55]. Furthermore, the ADAR1 p110 isoform has been discovered to possess a distinct function of preserving genomic stability through the catalyzation of R-loop structures (Figure 1D). These structures represent a stable form of the RNA/DNA hybrid on telomeres, which may sometimes occur when newly synthesized RNA molecules fail to detach from their template DNA immediately after transcription [86]. 

### 2.3. Regulatory Functions of Site-Specific dsRNA 

RNA editing sites are predominantly found in intronic, intergenic, and 3′UTR regions, with only a few in exons. These sites are known to affect RNA splicing and may also have an impact on miRNA-mediated transcript regulation, as well as altering the mRNA sequence, thus ultimately changing protein functions [9].

#### 2.3.1. Recoding RNA Editing

**Figure 2:**
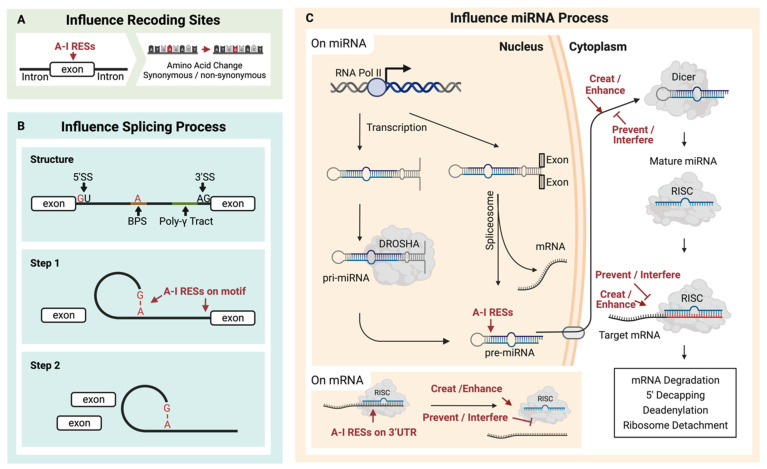
Multiple contributions can result from A-I RNA editing sites (RESs). (**A**) RESs located on exons may cause amino acid recoding. (**B**) RESs located on splicing motifs may change splicing events, and (**C**) RESs on pre-miRNA may affect the formation of miRNA and consequently impact downstream mRNA regulation induced by miRNA.

Recoding editing sites (Figure 2A) were widely found in diseases with cis [48] or trans [87] regulators. In cancers, RESs that result in non-synonymous substitution of codons could introduce proteomic diversities [88] to affect cancer cell proliferation, migration, and invasion, such as RES in the coding region of COPA. RES could also create neoantigens in cancer cells to promote the activation of the immune system [17]. 

#### 2.3.2. RNA Editing Influences RNA Splicing

ADAR-mediated A-I conversion could alter RNA splicing sites (Figure 2B). As a functional splicing element, the 5′-donor sequence is mostly conserved as GU, while the 3′-acceptor sequence is predominantly AG [89]. Additionally, the splicing element branch point sequence (BPS) contains an adenosine residue that will accept a guanosine residue at the 5′ end of the intron [89,90]. Because the inosine could work as guanosine, these elements might be destroyed [91] or created [92,93] by A-I RNA editing in pre-mRNA. As a result, the editing of pre-mRNA might contribute to multiple effects, such as exon skipping or novel exon [94]. Except for directly editing these elements, other relationships between A-I RNA editing and splicing should exist [95]. Kapoor et al. found that some splicing perturbations were provided independently of the editing process through physical interaction with the splicing machinery [96].

Knocking out ADAR1 and ADAR2 in mice experiments caused significant changes in splicing [96]. Most splicing modulation events are associated with ADAR1, while ADAR2 is one of two subforms of catalytic RNA-editing ADAR. These two editing enzymes are not replaceable, especially at some specific sites. For example, intron 42, which was retained in Filamine A, has been proven to be a specific target for ADAR2 editing, with little association with ADAR1 editing [97].

RNA editing can create alternative splicing isoforms that can be oncogenic. One example is ADAR1 editing, which can affect splicing through the edited site, while ADAR2 binding can prevent U2AF65 from entering the 3′ splice site and subsequently blocking the splicing [15]. Splicing changes regulated by ADARs have been reported not only as a by-product of ADARs editing but also in relation to tumorigenesis. The dysregulation of RNA editing and altered splicing has been reported in breast cancer, B-cell lymphoma, and other cancers [98,99,100]. Alternative splicing could also be tumor-suppressive. In cancer cells, the protoplasts of CCDC15 (including exon 9) were oncogenic, and ADAR acted as a tumor inhibitor by influencing the growth of cancer cells through binding or catalytic editing, which makes the exon skip [15].

RNA editing and splicing exhibit a mutually regulatory relationship. RNA editing precedes splicing in most transcripts [93]. In some cases, splicing regulates editing by affecting the conformation of splicing transcripts. For example, the osmosensitive cation channel TMEM63b has interdependent exon four skipping and an ADAR2-mediated recoding site in exon 20, Gln to Arg. Wu et al. found that if exon four is retained, it destroys the hairpin structure in exon 20, which is prone to ADAR2 action [101]. In addition, ADAR-mediated binding or catalytically regulated splicing may be flexibly regulated in homeostasis. A major splicing factor, SRSF9, inhibits specific editing on novel exons. SRSF9 is a major splicing factor that has been found to inhibit specific editing of some exons in the brain, such as voltage-gated calcium channel CaV1.3. The physical interaction between SRSF9 and ADAR2 could decrease the editing function by preventing the dimerization of ADAR2. In the two RNA recognition domains of SRSF9 (RRM1 or RRM2), RRM2 is mainly involved in the interaction between ARSF9 and ADAR2 [102,103]. 

#### 2.3.3. RNA Editing Regulates miRNA Binding

MicroRNAs are short RNAs of about 22 nucleotides that regulate gene expression [104]. The much longer miRNA precursors (pre-miRNAs) contain dsRNA and are potential substrates for A-I RNA editing (Figure 2C). Around 20% of miRNA precursors are edited in the adult brain [105]. It has been reported that several miRNA RESs conserved in ten different human tissues may increase the diversity of miRNA targets and regulate miRNA function [106]. RES on microRNA affects the processing of the miRNA. ADAR1 could form protein complexes with endoribonuclease Dicer. The catalysis-inactivated heterodimer promotes Dicer to recognize miRNA precursors, thereby increasing the miRNA processing to produce more miRNA. Meanwhile, the interaction between ADAR1 and Dicer could promote miR-155-5P maturation and further inhibit adipogenesis [107]. The catalysis-activated homodimers (ADAR1-ADAR1) edited microRNA to block the recognition and cleaving by the miRNA process enzyme, Dicer or Drosha, and thus reduce the miRNA process. In addition, the influence comes from protein–protein interaction. The RES on the miRNA could affect the multiple miRNA process stages, such as the recognition by the Dicer–DGCR8 complex, which has been proved for several miRNA precursors of pri-miR-142 [108], pri-miR-33, pri-miR-133a2, and pri-miR-379 [105]. In contrast, RES enhances the Drosha cleavage for pri-miR-197 and pri-miR-203, also reported by Kawahara et al. In addition, in human melanocytes, RES inhibited the Drosha on pri-miR-455 at the +2 and +17 positions, further decreasing miR-455-5p levels. The suppression of ADAR1 is linked to an increase in miR-455-5p, thus leading to a decrease in cytoplasmic polyadenylation element-binding protein 1 (CPEB1), a tumor suppressor. This association may be a contributing factor to melanoma metastasis [109]. Except for Drosha, RES also affects Dicer cleavage to inhibit miRNA expressions, such as pri-miR-151 [110] and pre-let-7g [105]. Additionally, RES on the precursor of miR-BART6-5p of the DNA virus, Epstein–Barr virus (EBV), can disturb the binding to AGO2-containing RISC, thus inhibiting this miRNA and blocking the specific binding to human Dicer mRNA [111].

A substitution in a single site on the pre-miRNA seed sequence can change the target mRNA, just like the RES in miR-376 alters the target specificity [112]. This can be significant in embryo development [113]. ADAR1 regulates miRNA-targeted mRNA by editing it, thus preventing miR-155 from binding to MDM2 3′ UTR. This results in the non-suppression of MDM2 and no activation of downstream p53 [14]. 

### 2.4. The Upstream Regulators of RNA Editing

Besides studying the functions of specific RNA editing sites, many studies aim to understand the global regulation of ADAR activity. RNA editing is known to introduce diversity in the transcriptome and proteome, but ensuring that its activity is regulated in living organisms is essential. Studies have shown that the efficiency and specificity of edited sites can be controlled. Proteins that bind to ADAR can affect its activity. These include all DZF-domain-containing proteins, such as ILF3, which can negatively regulate RNA editing [87]. 

The RNA editing site shows some specific primary sequence preferences. The 5′ neighbor of the sites mostly influences the catalysis of ADAR1 and ADAR2 [114,115,116]. The 3′ neighbor affects ADAR2 more. Thus, ADAR2 prefers certain trinucleotides, such as UAU, AAG, UAG, and AAU [115]. Based on the primary sequence, by learning the structural characteristics of the nearby sequence, there is the possibility of predicting the RESs [116]. Stabilizing the hairpin covering the RES influences the editing efficiency, and the decreased hairpin stabilization could reduce the editing frequency [46]. The cis-regulation is conserved in *Drosophila* species. Also, the earlier RESs in the species’ evolutionary tree tend to have higher editing efficiency, and these sites are enriched in neuronal genes [117]. The secondary structure of the substrate (hairpin or sense–antisense structure) could interfere with the access of the enzyme, and the binding of the enzyme would, in turn, open around ten base pairs for further catalysis [1]. In addition, tertiary structure in vivo could influence RNA editing efficiency [118]. Moreover, it is plausible that remote sequence structures may affect RNA editing events [119]. Quantitative trait loci analysis in *Drosophila melanogaster* showed that functional edQTLs may work by changing the secondary structure and affecting nearby RNA editing levels [1]. In addition, only a tiny fraction of dsRNAs must be edited to change their immunogenic secondary structure [120,121]. Sun and colleagues showed that immunogenic hairpins have shorter loops between stems than other dsRNAs [121].

In addition, other types of RNA modifications may affect ADAR-mediated RNA editing. The deamination activity of ADAR2 is sensitive to RNA modification at the 2′-carbon ribose. Methylation on 2′-OH would lead to a significant decrease in deamination efficiency, while 2′-deoxyadenosine and 2′-deoxy-2′-fluoroadenosine would not change the rate much [122]. The 2′-O-methylation on the ribose of eukaryote mRNAs 5′ cap could, similarly to ADAR-mediated self/non-self dsRNA, distinguish and avoid coronavirus by activating the MDA5-mediated IFN pathway [68].

The RNA editing sites often cluster closely in specific genome regions, especially in aging or diseased samples. This phenomenon is referred to as hyper-editing. Hyper-editing regions exist in the aging human brain, and these hyper-editing sites remove the loops in hairpins and make the dsRNA structure more stable [123]. Hyper-editing also occurs in the 3′UTR of MDM2 mRNA, and these RESs block the related miRNA, miR-155, and binding, which stabilizes the MDM2 mRNA and is finally involved in promoting the malignant progenitor propagation [14]. Emerging RES hotspots were further found in cancers. Hyper-editing sites block the recognition between miR-200b and the 3′UTR of ZEB1/ZEB2 mRNA, thus reducing the inhibition of ZEB1/ZEB2 to promote cell invasion and migration. Also, the hyper-edited miR-200b could target new mRNA, such as LIFR, a metastasis suppressor [124].

## 3. ADAR-Mediated RNA Editing in Homeostasis and Immune-Related Diseases

### 3.1. RNA Editing in Maintaining Homeostasis

RNA editing plays a crucial role in the widespread editing of MDA5-recognized dsRNA and the specific editing of sites that serve diverse functions. ADAR1 is vital for the survival of mouse embryos by deleting *Adar1*, which could result in a lethal IFN disorder. Meanwhile, ADAR2 is also necessary in mammals. The lack of ADAR2 may result in death with seizures in mice, but artificial single-site A-I editing of GluR2 pre-mRNA can rescue the condition by mimicking ADAR2 activity in vivo [125]. Although ADAR1 and ADAR2 edited an amount of overlapped RESs, these two proteins were not redundant in mice [126]. Notably, the tremendous difference in RES was observed in humans and mice mainly because of their different repeat elements [127]. No abundant Alu elements are present in mice, which are the primary substrate for ADAR1 in humans. Therefore, the animal experiment could not entirely identify the ADAR1 function in humans [50,127,128]. However, it is worth noting that most ADAR-mediated RNA editing is not vital for mammalian homeostasis because of the survival of the mouse with both knockouts of ADAR1 and MDA5 [126]. 

A series of efficient and highly specific RNA editing sites have been found in exons in healthy or diseased mammals [22,129,130], and these RNA editing sites are usually formed by pairing exons with nearby introns in a sense–antisense structure [131]. It is important to note that RES exhibits strong tissue specificity, thus forming distinct RES clusters specific to various human body tissues. Analysis of Genotype-Tissue Expression (GTEx) data shows that 3710 sites exist primarily in only one of these tissues [45]. Within coding regions, single-cell RNA-seq data revealed that 1517 RESs were identified and exhibited significant differences in editing levels across human tissues, thus contributing to protein diversity among cell subpopulations [130]. The cells related to the brain and nerves exhibit the highest ADAR editing in human beings, while RNA editing is remarkably low in skeletal muscles. In comparison to the brain, the arteries, colon, and esophagus are much more likely to undergo recoding RNA editing. However, the recoding sites of these tissues are mainly in a small number of targets with high editing frequency (especially FlnA and IGFBP7), while editing in the brain is more diverse and affects more target genes. Although RESs vary significantly between tissues, clustered RESs across species can be clearly distinguished by species [45]. In a genome-wide association study of inflammatory diseases, 30,319 cis-RNA editing quantitative trait loci (cis-edQTLs) were identified in 49 human tissues [22], partially explaining the genetic basis of RNA editing sites. The number of cis-edQTLs shows high variabilities among different tissues, from a few hundred to a few thousand. The highest number of cis-edQTLs in the thyroid is close to 10,000. In the nerve (tibial), it is close to 8000. Meanwhile, in lymphocytes, it is only around 1000 to 2000. 

Most research on ADAR’s ability to adjust to dynamic environmental conditions is carried out using animal models. Nevertheless, these experiments are also relevant to ADAR’s role in humans. *Octopuses* could utilize specific RESs to alter kinesin motility and calcium-binding affinity of synaptotagmin to quickly adapt to water temperature shifts with a rapid increase in the global editing level [132]. Garrett and Rosenthal also reported a recoding site editing of *Octopuses* on an ion channel to increase the speed of gating kinetics to adapt to environmental temperature shifts [133]. The temperature-dependent RES changes were also detected in *Drosophila* in different living temperatures or when facing an acute temperature change [134,135,136]. 

In humans, the hippocampus-specific low-edited glutamine site on GluR-B (an AMPA, α-amino-3-hydroxy-5-methyl-4-isoxazole propionate, receptor), GluR-B Q/R site, was found in Alzheimer’s disease. In ADAR2-/- mice, a similar lethality and seizure phenotype resulted from the under-editing of this site [125]. The calcium permeability of AMPA receptors (AMPARs) in mammalian brain excitatory neurons could be blocked by a co-assembly of GluR-B subunits whose arginine (R) residues at crucial sites in the channel pore could be created by ADAR2-mediated selective adenosine deamination of glutamine (Q) at the pre-mRNA level at more than 99 percent [125,137,138,139]. In most of the 24 RESs across different transcripts, a significant reduction was observed in editing frequencies in ADAR2-/- mice. However, it is important to note that these editing changes are not considered the leading cause of symptoms, and how they function and can be controlled is still unknown [125]. In addition, in relation to human neural development, Chen and Yang proposed that ADAR1 regulates the differentiation of human embryonic stem cells and affects neural formation through miRNA interaction in an editing-independent manner [140]. Moreover, editing could also affect cell renewal and differentiation, such as in the case of the RES on the 3′UTR of AZIN1 [141].

Generally, for global patterns in mouse models, knockdown of ADAR1 results in early death, but it can be rescued by knockout of the MDA5 pathway. Knockout of ADAR2 causes seizures and premature death, which can be rescued by introducing Q/R editing sites on GluR-B. Apart from inhibiting the immune pathway caused by immunogenetic dsRNA, another key role of ADAR may be related to site-specific editing in most sites, which could be ineffective in maintaining homeostasis. Therefore, ADAR enzymes play a significant role in modifying the editing frequency at specific sites impacting cellular homeostasis. Of course, changes in the global level of ADAR editing may also indicate the disease status.

### 3.2. Targeting Dysregulation of RNA Editing in Immune-Related Diseases

#### 3.2.1. Multiple Roles of ADAR-Mediated RNA Editing in Cancers

**Figure 3:**
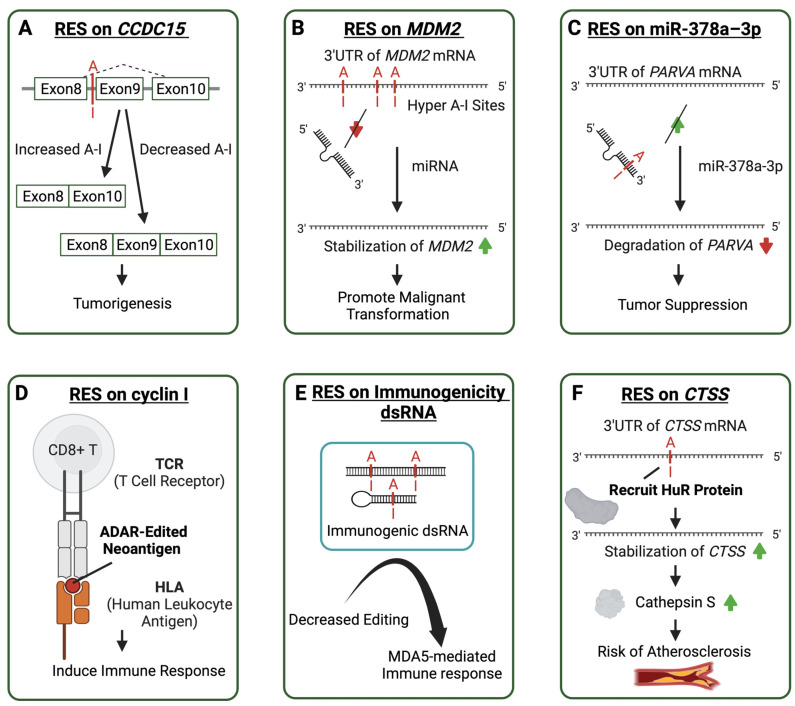
Molecular mechanisms of aberrant ADAR-mediated RNA editing lead to the pathogenesis of multiple diseases. Cases of ADAR editing sites contribute to immune-related diseases in various ways. A-I editing sites are shown in red. Arrows in red represent decrease and green arrows represent increase. (**A**) An A-I editing site affects the splicing of Coiled-Coil Domain Containing 15 (CCDC15) related to tumorigenesis. (**B**) Multiple A-I editing sites on the 3′ untranslated region (3′UTR) of Mouse Double Minute 2 (MDM2) prevent microRNA binding to promote malignant transformation. (**C**) A-I editing on the microRNA increases its affinity to the 3′UTR of Parvin Alpha (PARVA) to suppress tumors. (**D**) ADAR editing creates neoantigen in cyclin I, resulting in an induced immune response. (**E**) Decreased editing on the immunogenic dsRNA in autoimmune diseases leads to an MDA5-mediated immune response. (**F**) The RNA editing on the transcript 3′UTR of the catherpsin S protein recruits the HuR protein to make the transcript more stable and encode more catherpsin S, thus increasing the risk of atherosclerosis.

Site-specific RNA editing is associated with various immune-related complex diseases. In human cancers, RNA editing abnormalities are strongly associated with high aggressiveness and poor prognosis in many malignant tumors. ADAR was considered to affect the immune microenvironment, such as by stimulating T lymphocytes and promoting the presence of more abundant M1 macrophages [142]. Many specific sites have been identified to play roles at different stages of cancer, such as tumor-promoting RES on the intronic region of the CCDC15, which affects the splicing process. In addition to influencing the structure of RNA during splicing in a catalytically independent or non-independent manner, the effect of ADAR on splicing can also be achieved in an SR protein-dependent manner. In vitro cell experiments with point mutations have demonstrated that the degree of editing of a RES upstream CCDC15 exon9 enhances the binding of SR protein, thus repressing the exon9 inclusion and producing a potentially oncogenic CCDC15 isoform [15] (Figure 3A). RNA editing also often occurs in the 3′ UTR of mRNA. Multiple RNA editing sites on the 3′UTR of the MDM2 (Figure 3B) stabilize its mRNA by decreasing regulatory miRNA binding, and over-existing MDM2 protein could enhance the propagation of the blast crisis progenitor, which might be involved in the malignant transformation of progenitors [14]. Cancer-associated RNA editing sites are also commonly found on regulatory miRNAs. ADAR1 could also be tumor-suppressing by introducing RES on miR-378a–3p (Figure 3C) to preferentially bind to the 3′UTR of oncogene PARVA, thus inhibiting its expression and preventing melanoma progression [16]. Also, recoding RES could create neoantigens to induce an immune response, such as RES on cyclin I. Peptides resulting from RNA editing can be presented by human leukocyte antigen molecules. In cell-based experiments, overexpression of the edited cyclin I protein can even lead to the cytotoxicity of tumor-infiltrating lymphocytes (Figure 3D) [17]. Most particular RESs were found to be specific to different types of cancer and lacked robust universal features across multiple cancer types [143]. Oncogenic recoding site S to G in AZIN1, which participates in cancer growth, invasion, and migration, was detected in various cancers, such as liver hepatocellular carcinoma (LIHC), esophageal squamous cell carcinoma (ESCC), non-small cell lung cancer (NSCLC), and colorectal cancer (CRC) [144,145,146,147].

Except for non-synonymous substitutions on coding sequences (CDS) [17], other types of editing sites, such as those on 3′UTRs (on GM2A) [19], Intron (FAK) [13], miRNA (miR-222) [20], long non-coding RNA (PCA3) [148], and circular RNA [149], have also been implicated in the progression of cancer. In addition, RES could improve anti-tumor drug resistance, such as by editing the 3′UTR of DHFR [150] and a recoding editing site on GLI1 [18]. Another study conducted a single-cell sequencing analysis of lung adenocarcinoma and found that cancer cells undergo more ADAR-like RNA editing than other cells. In the single-cell RNA sequencing data of tumors and adjacent tissues from 30 non-small cell lung cancer patients, an average of 672 sites were edited per cell and 50,576 RESs in at least 50 cells [151]. Furthermore, genes associated with drug treatment were enriched with RESs, suggesting that ADAR-mediated RNA editing may contribute to drug resistance in lung cancer [151]. RES on miR-378a-3p [16] and miR-455-5p [12] could prevent or promote tumor metastasis. Martínez-Ruiz et al. indicated that the transcriptional editing pattern of metastatic tumor cells originated from the primary tumor, thus showing a possibility of gaining continued evolution [152].

#### 3.2.2. Immunogenic dsRNA RNA Editing may Regulate Autoimmune Diseases

A recent genotype–phenotype association study using GTEx (27 normal human tissue types from GTEx v7) and Geuvadis (64 immortalized B cell lines) sample data found that human A-I RNA editing sites are enriched in genome-wide association study (GWAS) signals for autoimmune diseases, such as Crohn’s disease, inflammatory bowel disease, and asthma [21]. Another study identified 30,319 cis-RNA-edited QTLs (edQTLs) in 49 human tissues. The study found that common genetic variants associated with RNA editing levels were significantly enriched in GWAS signals for common inflammatory diseases and that genetic risk variants associated with GWAS were generally associated with reduced levels of nearby dsRNA editing [22]. Decreased RNA editing sites on putatively immunogenic dsRNAs, including inverted-repeat Alu elements and cis-natural antisense transcripts, could induce MDA5 activation and therefore increase the immune response in autoimmune diseases (Figure 3E) [79].

In the meantime, research has shown dysregulation of ADAR-mediated RNA editing in multiple autoimmune diseases. In rheumatoid arthritis patients, ADAR1 was significantly overexpressed in the synovium, and the expression of the ADAR1 p150 isoform in the blood was significantly increased, with a significant increase in A-I RNA editing. ADAR1 p150 expression and individual adenosine RNA editing rates for cathepsin S *AluSx*^+^ decreased in patients with an excellent clinical response [153]. Blood samples from patients with systemic lupus erythematosus also have high levels of RNA editing, some of which affects proteins and may produce new autoantigens, thus increasing the antigen load [154]. In patients with primary Sjögren’s syndrome, there was a significant increase in overall RNA editing associated with ADAR1 p150. The top 10 differential editing sites were found in 9 unique genes involved in the inflammatory response or the immune system, and these RESs can potentially be disease-specific biomarkers [155].

#### 3.2.3. RNA Editing in Other Immune-Related Diseases

In cases of schizophrenia, a neuropsychiatric disorder that may be caused by abnormalities in the immune system [156], researchers have identified two adjacent and interrelated RNA editing sites on the MFN1 protein. MFN1 encodes a mitochondrial membrane protein that is necessary for mitochondrial fusion. One of these sites is a C-T site, which APOBEC3B could edit, while the other is an A-G site that ADAR could edit. These editing sites are conserved in multiple species, such as macaques, mice, and zebrafish. Mouse models have shown that both of these recoding sites in Mfn1 could reduce the ability of MFN1 in mitochondrial fusion, which could lead to defective cellular responses to adaptive stress. At the same time, these two editing sites could also increase the level of apoptosis. The editing levels of MFN1 C-T and A-G are correlated, and dual editing could lead to a higher degree of mitochondrial fusion and apoptosis [23]. 

In patients with immune-related atherosclerotic vascular disease, the degree of RES editing on the 3′UTR of cathepsin S (*CTSS*) is related to the ADAR1 level. The altered editing degree may regulate cathepsin S expression level by recruiting HuR protein to the 3′UTR of *CTSS* to stabilize the mRNA and increase the encoding level of cathepsin S to participate in the occurrence and development of atherosclerosis (Figure 3F) [157].

#### 3.2.4. Therapeutic Implication of ADAR-Mediated RNA Editing

In recent years, by regulating the global RNA editing level, research has increasingly indicated the therapeutic potential of ADAR-mediated RNA editing in treating human cancers. The silence of ADAR in mouse models can transform cold tumors into hot tumors by activating interferon-associated immunity in the tumor microenvironment, and reducing ADAR expression in tumors could increase sensitivity to checkpoint inhibitors in melanoma mouse models [158]. ADAR silent treatment could bypass the typical resistance of small-molecule anticancer drugs against the immune checkpoint blockade (ICB), such as the PD-1 checkpoint blockade, in tumor molecules together with PKR and MDA5 activation, thus resulting in tumor cell growth inhibition and inflammation [59,159,160,161].

ADAR-related tumor therapy could also rely on the ZBP1 pathway inhibited by ADAR. A small molecular curaxin CBL0137 was found to activate the immune pathway by triggering the formation of Z-RNA, which could induce ZBP1-mediated necrosis in cancer-associated fibroblasts in a mouse melanoma model [59].

In addition, complete or partial inhibition of ADAR1 may enhance the apoptosis and/or cytotoxic effects of splicing inhibitors in anticancer therapy. Dysregulated ADAR-mediated RNA editing and RNA splicing are both commonly observed in many cancers [94]. In an MYC-driven triple-negative breast cancer model, spliceosome-targeted therapy (STT) has been shown to induce aberrant RNA splicing to produce immunogenic mRNA. By accumulating mis-spliced mRNA containing large amounts of endogenous dsRNA in the cytoplasm, downstream immune and tumor cell apoptosis would be stimulated [162]. Inhibition of ADAR1 likewise increases the accumulation of dsRNA to activate type I interferons, and a synergistic effect between ADAR1 inhibition and STT has been shown to increase the effect [163]. Further research is necessary to better understand the contribution of ADAR in STT.

## 4. Emerging Computational Resources for RNA Editing Data Analysis

### 4.1. Characterizing RNA Editing from High-Throughput Sequencing Data

**Figure 4:**
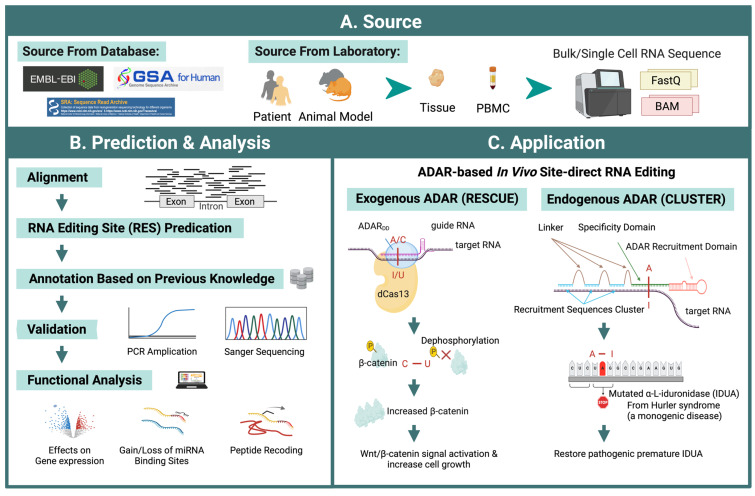
Workflow of ADAR editing site prediction and potential therapeutic application. (**A**) Data sources can be obtained from publicly available RNA-seq databases or from sequencing in-house samples from human and animal models. (**B**) Predicting RNA editing sites from RNA-seq data involves multiple steps to increase accuracy. Several databases collect RNA editing sites that can be used for prediction reference and RES annotation. The function of these predicted editing sites can be analyzed using downstream methods, such as differential modification, regulatory impact on gene expression, gain/loss of miRNA binding, or peptide recoding. Some of these analyses can be performed via interactive web servers. (**C**) ADAR-based in vivo site-direct RNA editing could be classified into two types. Methods based on exogenous ADAR, such as RESCUE, as an example, using modified ADARdd (the adenine deaminase domain of ADAR2 was evolved) fused with catalytically inactivate RNA-targeting CRISPR-Cas13 (dCas13) delivered by designed vehicles to edit target RNA sites. RESCUE has been used to dephosphorylate β-catenin through a C-U conversion, thus leading to T41I substitution and resulting in β-catenin accumulation and increased Wnt/β-catenin signal and cell growth. Endogenous ADAR, such as CLUSTER, was tested on mutated IDUA, which recovers a premature stop codon through A-I editing. A-I RNA editing or C-U RNA editing are shown in red.

Identification of RNA editing sites for high-throughput RNA sequencing data has become a popular approach due to the increasing recognition of the importance of global or site-specific RNA editing. A large number of available data sources are provided by large database resources and more convenient sequencing methods in the laboratory (Figure 4A). Over the past two decades, various computational tools for predicting RNA editing sites have been developed with multiple methods to increase accuracy and efficiency (Table 1). Raw high-throughput RNA sequencing data first undergo quality control, which usually dumps or trims low-quality reads, thus yielding high-quality data for subsequent reads aligning on the reference genome, such as hg38 for humans. Then, RNA editing sites could be identified as one specific type of genomic mutation indicated by annotating a single base substitute on high-quality mapped reads (Figure 3). The precise RES detection is the foundation of the subsequent analysis of the RES functions. A series of software take multiple strategies to filter out false-positive sites and could be classified into several categories. Various tools [25,164,165,166,167,168] adopt approaches like statistical modeling or machine learning techniques, such as the Generalized Linear model (L-GIREMI), random forest classifier (RDDpred), Logistic Regression (RED-ML), and Support Vector Machine (RDDSVM), to increase the sensitivity of detecting RESs. Others further used deep learning to increase detection accuracy [26,169]. Other tools design specific mapping schemes for repetitive genomic regions to increase the accuracy of RES detection [170]. Some tools primarily focus on hyper-editing sites to further characterize RESs that cannot be detected from the typical mutation detection method [171,172]. These tools use unmapped reads to call clusters with hyper-editing via the global A-G mask with a series of filtrations. Some tools are advantageous in identifying reads with spliced junctions [170]. 

Methods use different strategies to reduce false positives caused by mapping artifacts. In the case of JACUSA, sequence features will be numerically included to filter out false positives caused by sequencing [165]. Although RES prediction tools are becoming more accurate, it is still recommended to experimentally validate them through PCR amplification or Sanger Sequencing before further functional analysis of essential RESs. (Figure 4B). Differential RNA editing analysis between disease and control samples is usually performed before subsequent functional analysis. To perform differential RNA editing analysis, most studies adopt a nonparametric Wilcoxon rank sum test of editing frequencies of RESs between groups [11,173,174], while a few approaches are based on modeling of editing levels or discrete NGS read counts [173].

**Table 1:** Computational tools for RNA editing site (RES) prediction and analysis.

Tool	Feature	Model	Programming Language	Input Format	URL [Accessed on 12 December 2023]
AEI [174]	Global editing level on the Alu element.		C	bam	https://github.com/a2iEditing/RNAEditingIndexer
DeepEdit [26]	Detect RNA editing site (RES) on a single Nanopore read.	Neural network model	Python	fastq	https://github.com/weir12/DeepEdit
DeepRed [169]	Predict RES using primitive RNA sequences.	Deep learning	MATLAB		https://github.com/wenjiegroup/DeepRed
JACUSA v1.2.0 [165]	Filter RES with sequence characteristics.		Java	fastq	https://github.com/dieterich-lab/JACUSA
L-GIREMI v0.1.12 [25]	Detect RES on a single long read. Detect rare allele-specific RES. Detect hyper-edited Alu. Predict RES-altered RNA secondary structure.	Generalized linear model	Python	bam	https://github.com/gxiaolab/L-GIREMI
RASER v0.5.2 [170]	An accurate read aligner with novel mapping schemes and index tree structure.		C++	fastq fasta	https://github.com/jaegyoonahn/RASER
RDDpred [164]	Distinguish between false-positive sites in RNA editing events.	Random forest classifier	Python	bam	https://github.com/vibbits/RDDpred
RDDSVM [168]	Use experimentally verified RES to predict novel RES.	Support Vector Machine	R		https://github.com/huseyintac/RDDSVM
RED-ML [166]	Detect new RES with confidence scores in a user-friendly way.	Machine learning	C	bam	https://github.com/BGIRED/RED-ML
REDItools v1/2 [24]	Characterize large-scale RES in dataset repositories, such as TCGA or GTEx.		Python	bam	https://github.com/BioinfoUNIBA/REDItools2
RES-Scanner [175]	Infer genomic locus genotype.	Bayesian model	Perl	bam fastq	https://github.com/ZhangLabSZ/RES-Scanner
RESIC [172]	Integrate detection and classification approaches into a pipeline. Identify hyper-edited regions.		Python	fastq	
SPRINT v0.1.8 [171]	Hyper-RES detection.		Python	bam	https://github.com/jumphone/SPRINT

### 4.2. Web Resources for RNA Editing Site Collection and Functional Analysis

Extensive sequencing of human tissues and animal models has resulted in the publication of numerous databases containing information regarding A-I editing sites. This information is highly beneficial for conducting research on specific sites (Table 2). Most of these come from healthy human samples and animal models, such as mice, fruit flies, pigs, and rhesus. For instance, RADAR and REDIportal are widely used RES databases that generate site information from the RNA sequencing data [28,53]. DbRES is a database covering 96 organisms, such as plants, metazoans, protozoa, fungi, viruses, etc., with 5437 RNA editing sites and 251 transcripts [176]. Considering the tissue-specific characteristics of A-I RNA editing, most databases allow for separate querying and analysis of RESs from different tissues. Apart from describing the tissue-specific distribution of editing sites, other data types, such as genomic mutations and cross-species conservations, were included to classify and explore the genetic basis of editing sites [22,29,177,178,179]. Other databases focus on RESs on coding regions, microRNAs (miRNAs), or long non-coding RNAs (lncRNAs) [27,180,181,182]. To uncover the potential clinical impact of RES, researchers are building data resources covering editing sites associated with human diseases, such as cancer and inflammatory disease [11,22,29,177,183,184]. Differential RNA editing analysis between disease and control samples is usually performed before subsequent functional analysis. To perform differential RNA editing analysis, most studies adopt a nonparametric Wilcoxon rank sum test of editing frequencies of RESs between groups [11,173,174], while a few approaches are based on modeling of editing levels or discrete NGS read counts [173]. In addition, there are also some interactive web servers published for functional interpretation and visualization of RESs [183,184,185].

**Table 2:** Databases and web servers for documenting and analyzing RNA editing sites (RESs).

Database	Description	RES Amount	Species	URL
dbRES [176]	Collection of known RNA editing sites with comprehensive annotations.	5437	96 species covering plant, metazoan, protozoa, fungi, and virus	http://bioinfo.au.tsinghua.edu.cn/dbRES [Accessed on 12 December 2023]
e23D [27]	Database of RES mapped to evolutionary-related 3D protein structures.	2,576,459 (human) 8823 (mouse) 5025 (fly)	Human Mouse Fly	
GPEdit [29]	Collection of RNA Editing quantitative trait loci (edQTL) in cancers.	320,029 (edQTLs)	Human (33 cancer types)	https://hanlaboratory.com/GPEdit/ [Accessed on 12 December 2023]
LNCediting v1.0 [181]	RES in lncRNAs with their effects on lncRNA secondary structures and lncRNA–miRNA interactions.	199,991 (human) 1922 (mouse) 165 (rhesus) 1829 (fly)	Human Mouse Rhesus Fly	http://bioinfo.life.hust.edu.cn/LNCediting/ [Accessed on 12 December 2023]
miR-EdiTar [180]	Predicted A-I-edited miRNA binding sites.	10,571	Human	
miREDB [182]	RNA editing on miRNAs.	4162 in around 80% of pre-miRNAs and 574 in mature miRNAs	Human Mouse Drosophila	
PRES [185]	Web server for downstream functional perturbations at RES.	-	Human	http://bio-bigdata.hrbmu.edu.cn/PRES/ [Accessed on 12 December 2023]
PRESDB [179]	Pig genome-wide RNA-editing investigation.	59,472	Pig	https://presdb.deepomics.org/ [Accessed on 12 December 2023]
RADAR v1/2 [53]	Collection of RESs, including tissue-specific editing levels.	Humans (1,379,403) Mouse (8108) Drosophila (2698)	Human Mouse Drosophila	http://rnaedit.com/ [Accessed on 12 December 2023]
REDIportal v1/2 [28,186]	ATLAS of RESs in human tissues and other organisms.	16 million (human) 107,094 (mouse)	Human (31 tissues) Mouse (2 tissues)	http://srv00.recas.ba.infn.it/atlas/ http://srv00.recas.ba.infn.it/atlas/index.html [Accessed on 12 December 2023]
REDR [187]	Potential regulation of RNA editing in drug resistance to 18 anticancer drugs.	7157 DESs from 98,127 informative RESs	Human (6 cancer types)	http://www.jianglab.cn/REDR/ [Accessed on 12 December 2023]
REIA [183]	Interactive web server that analyzes and visualizes RESs in cancers.	8,447,588	Human (34 cancer types)	http://bioinfo-sysu.com/reia [Accessed on 12 December 2023]

## 5. Application of Endogenous ADAR-Mediated Precise RNA Editing

RNA editing enzymes have been used to create site-direct RNA editing tools based on recent years of interpretation of ADAR substrate sequences and understanding of RNA editing at specific sites (Table 3). Reversible RNA editing in living cells has excellent application prospects in treating diseases and studying RNA or proteins. Unlike DNA editing, RNA editing does not introduce mutations directly into the genome but edits the transcribed RNA to gain a reversible and dose-dependent way to manipulate genetic information. Such properties make RNA editing significantly safer in gene-drug development [188]. 

Site-directed RNA editing tools can be categorized into two types: those that employ exogenous ADARs and those that recruit endogenous ADARs. ADAR2 is typically used in the former, while both ADAR1 and ADAR2 are recruited for editing in the latter [189]. In the SDREs using exogenous ADAR, to increase the precision of the editing, Cas13 [190] was introduced as a guide system. Because the deaminase domain of ADAR2 could be relaxed to accept other bases to possess cytidine deamination activity, it could be extended for C-to-U RNA editing, which allows RNA editing to target more sites and enable the regulation of post-translational protein modifications, such as phosphorylation, to regulate residues associated with phosphorus signaling. On the transcript of β-catenin (CTNNB1), dCas13 and exogenous ADAR could increase ACC to AUC editing, thus resulting in T41I. T41I is located at a critical phosphorylation site, which could prevent phosphorylation from proceeding normally. As a result, β-catenin would increase and lead to nearly five-fold activation of Wnt/β-catenin signaling, ultimately leading to faster cell growth (Figure 4C) [32].

Most endogenous SDREs recruit ADAR1 or ADAR2 to catalyze. Methods improve the recruitment of ADAR by adding hairpin structures and R/G domains to guide RNAs. In Hurler syndrome, a monogenetic disease, CLUSTER was tested on mutated Alpha-L-iduronidase (IDUA). By recovering a premature stop codon through A-I editing, the expression of IDUA could be repaired (Figure 4C). By using endogenous ADAR to encode effecter RNA in the cell that is expressing cell-specific target RNA, cell classification and manipulation could be operated, such as the cellREADR [31,191].

Moving RNA editing to the clinic is still a challenging task. Firstly, the efficiency of RNA editing needs improvement [30,189]. Secondly, the current RNA tools prefer specific substrate sequences, which limits their practical applications. More breakthroughs are required to overcome these limitations and extend the scope of substrates [189]. Additionally, editing accuracy is a significant challenge, as current methods can only partially eliminate off-target editing [190,192]. For editing based on exogenous ADAR, there is also the issue of trafficking in vivo [189,192]. Improving editing tools is not the only challenge in RNA editing. Another challenge is selecting targeted sites. For diseases, the genetic causes of occurrence are often complicated and involve adaptive drug resistance or pathway bypass. Therefore, RNA editing still needs a lot of development before it can be widely applied in clinical settings. 

**Table 3:** Collection of site-directed RNA editing tools.

Name	Year	ADAR Source	Description
AD-gRNA [193]	2017	Endogenous	Use reprogrammable antisense region to target specific RNA sites and a hairpin structure on the guide RNA to recruit hADAR2.
Novel guideRNA [194]	2017	Endogenous	Use R/G-guide RNAs as trans-acting guide RNA.
REPAIR	2017	Exogenous	Catalytically inactive Cas13 (dCas13) is fused to the ADAR2 to edit.
SNAP-ADAR [195]	2018	Exogenous	SNAP-tagged ADARs with chemically stable guide RNAs allow simultaneous editing in multiple target transcripts with high efficiency and lower off-target rates.
CIRTS [196]	2019	Exogenous	An all-human protein RNA editing tool.
RESTORE [33]	2019	endogenous	Combine an ADAR recruitment domains (R/G motif) and a chemically modified guide region.
LEAPER [197]	2019	Endogenous	Recruit ADAR1 or ADAR2 through short-engineered ADAR-recruiting RNAs (arRNAs).
RESCUE [32]	2019	Exogenous	Programmable C-to-U RNA editing using ADAR2 fused to CRISPR-Cas13.
miniCas13X-ADAR2dd [198]	2021	Exogenous	Use mini Cas13X.1 protein to efficiently target RNA for A- I and C-U editing.
shAD-gRNA [199]	2021	Exogenous/ Endogenous	Use shAD-gRNA to have as short a sequence as possible to induce editing activity.
CellREADR [191]	2022	Endogenous	Utilize ADAR-mediated RNA editing to translate effector proteins in cell with the target RNAs.
CLUSTER [30]	2022	Endogenous	Utilize the recruitment sequence and R/G-binding domain.
cadRNA [200]	2022	Endogenous	Use circular ADAR recruitment guide RNA (cadRNA).
LEAPER 2.0 [192]	2022	Endogenous	Use covalently closed circular ADAR-recruiting RNAs (circ-arRNAs).
RADAR [31]	2023	Endogenous	RNA sensing in living cells using ADAR editing.

## 6. Conclusions

The ADAR enzyme catalyzes Adenosine to Inosine (A-I) conversion, a hallmark of RNA modification in metazoans via double-stranded RNA (dsRNA) deamination. Metazoans adopt A-I RNA editing for various physiological activities, such as adaptive mechanisms in *Octopuses* and *Drosophila* in response to changing environments. Although no RNA editing mechanism that responds to the environment has been identified in humans, RNA editing exhibits complex patterns in various human diseases, such as global editing landscape alterations in cancer and autoimmune diseases. ADAR-like RNA editing has a distinct regional preference, with most sites distributed on introns and 3′UTR, especially on Alu elements (one of SINE, short interspersed nucleic elements), which would form hairpins with dsRNA. The aberrant functioning of ADAR may result in either an escalation or reduction of the editing sites on a global scale. Such alterations can consequently impact the immune pathways, such as MDA5 or PKR-mediated pathways. Also, the ADAR1 p150 solely binds to endogenous Z-RNA, which is necessary to achieve cellular homeostasis by preventing the antivirus-like apoptosis pathways induced by the binding between unbound Z-RNA and ZBP1. 

Apart from globally distributed immunogenic dsRNA or hairpin editing, a site-specific RNA editing site could also directly impact protein functions. For instance, a recoding site located on an ion channel protein can control the rate of ion passage [132,136]. This highlights the significance of RNA editing in maintaining cellular homeostasis and preventing diseases. A-I RNA editing may affect protein degradation and exon splicing by disrupting microRNA interaction with 3′UTR and blocking splicing complex formation. It is worth noting that ADAR-mediated editing may have varying effects in different diseases. On the one hand, dsRNA editing ameliorates the immune response in autoimmune diseases. On the other hand, it can be oncogenic by introducing an immune-suppressive microenvironment in human cancers. ADAR can be a potential target in the development of anti-tumor therapy. Inhibiting ADAR can help prevent cancer cells from becoming resistant to anti-tumor drugs. Additionally, converting endogenous RNA to ADAR-target Z-RNA may increase the amount of unbound Z-RNA, which can stimulate cancer cell apoptosis. In addition to regulation at the overall ADAR editing level, modification at the editing level at specific sites can also be exploited as a potential treatment for diseases, such as T41I on beta-catenin and exon inclusion on *CCDC*. According to validation experiments and analytic methods, such as GWAS-edQTL colocalization and Mendelian Randomization, some key editing sites may have a causal relationship with specific diseases. 

It is important to note that RNA editing sites in mammals are dynamically changing, and such changes are regulated under environmental stress. A linkage reaction has been observed in two nearby sites in schizophrenia, one of which was edited by a DNA editing enzyme, APOBEC. In contrast, the other site was edited by ADAR [23]. This finding may suggest that the change in these two sites is under non-randomly adaptive selection. Furthermore, it has been discovered that ADAR-mediated editing is under stress, and a series of sites are specially edited with cis or trans regulators. This phenomenon indicates that other forms of RNA modifications in certain diseases, such as epigenetic or RNA secondary structure changes, lead to specific sites being edited. By exploring the linkage effect of editing sites under disease conditions, it may be possible to determine the importance of specific RNA editing sites. 

Based on the knowledge of ADAR and with the advancements in sequencing technology, it is now possible to fully characterize the whole spectrum of specific RNA editing sites in many immune-related diseases. However, it is crucial to exercise caution when interpreting RNA editing sites, particularly recoding sites, from sequencing data. This is because current analytical methods are susceptible to a high false-positive rate [201]. If these differentially edited sites in diseases are experimentally validated, they could potentially be targeted for engineered RNA editing as a therapeutic approach. Unlike DNA editing, engineered site-directed RNA editing is more morally sound and biologically safe in medical applications. Recently, site-directed methods for RNA editing in living organisms have been developed, thus opening new possibilities for RNA therapeutic approaches. RNA-based therapeutics have proven effective in targeting multiple cells or organs to restore genetic function, and many of them are already FDA-approved [202]. A recent study indicated that an AAV-mediated RNA editing tool with improved efficiency and specificity is viable for Hurler syndrome treatment in a humanized mouse model [203]. In conclusion, site-directed RNA editing holds considerable promise in the clinical application of therapeutic diseases, though many challenges remain to be resolved.

## Data Availability

Not applicable.

## Abbreviations

AGO2	Argonaute RISC Catalytic Component 2
APOBEC3B	Apolipoprotein B MRNA Editing Enzyme Catalytic Subunit 3B
AZIN1	Antizyme Inhibitor 1
Blcap	BLCAP Apoptosis Inducing Factor
CCDC15	Coiled-Coil Domain Containing 15
COPA	COPI Coat Complex Subunit Alpha
CyFip2	Cytoplasmic FMR1 Interacting Protein 2
DHFR	Dihydrofolate Reductase
FAK	focal adhesion kinase
FlnA	Filamin A
GLI1	GLI Family Zinc Finger 1
GM2A	Ganglioside GM2 Activator
IGFBP7	Insulin Like Growth Factor Binding Protein 7
ILF3	Interleukin Enhancer Binding Factor 3
ILFR	LIF Receptor Subunit Alpha
MDM2	Mouse Double Minute 2
MFN1	Mitofusin 1
MYC	MYC Proto-Oncogene, BHLH Transcription Factor
PARVA	Parvin Alpha
PCA3	Prostate Cancer Associated 3
RISC	RNA-induced Silencing Complex
SRSF9	Serine And Arginine Rich Splicing Factor 9
TMEM63b	Transmembrane Protein 63B
U2AF65	U2 small nuclear ribonucleoprotein auxiliary factor 65
ZEB1/2	Zinc Finger E-Box Binding Homeobox 1/2
